# Covid-19 Coping Survey: an In-depth Qualitative Analysis of Free-Text Responses from People With and Without Existing Health Conditions in the UK

**DOI:** 10.1007/s12529-022-10055-z

**Published:** 2022-02-07

**Authors:** Rachael M. Hewitt, Judith Carrier, Stephen Jennings, Lilith Nagorski, Rachael Pattinson, Sally Anstey, Rhian Daniel, Chris Bundy

**Affiliations:** 1 School of Healthcare Sciences, College of Biomedical & Life Sciences, Cardiff University, Eastgate House, 35-43 Newport Road, Cardiff, CF24 0AB UK; 2 Patient and Public Involvement co-investigator, Cardiff, UK; 3 Division of Population Medicine, Cardiff University, Heath Park, Cardiff, CF14 4YS UK

**Keywords:** SARS-CoV-2, Existing health conditions, Coping, Qualitative

## Abstract

**Background:**

There is currently a lack of qualitative research exploring how cognitive and emotional reactions to the threat of SARS-CoV-2 affected the health behaviours of people living with and without pre-existing mental and physical health conditions. We aimed to investigate how the threat of SARS-CoV-2 influenced the thoughts, feelings and health behaviours of people with and without pre-existing health conditions in the UK.

**Methods:**

A cross-sectional online survey of UK adults (aged 18 and over). Free-text responses were analysed using a qualitative framework approach guided by the Common-Sense Model of Self-Regulation.

**Results:**

Of the 9110 respondents, 2763 participants provided at least one free-text response. Three main themes were derived from the data. Theme one, *locus of control*, reports on the extent to which people felt in control during the first wave of the pandemic. Theme two, *emotional impact*, conveys how individuals felt and how people’s personal circumstances made them more vulnerable to experiencing negative emotions during the pandemic. Theme three, *coping strategies*, describes common health-protective and health-threatening behaviours performed by individuals, as well as the importance of social connectedness, the social context and the need for collective action during the first national lockdown.

**Conclusion:**

Complex psychological interventions including behaviour change are required to mitigate the psychological burden of the SARS-CoV-2 pandemic and increase autonomy in people with and without pre-existing conditions during this highly uncertain time. Behavioural scientists can support governments and public health agencies to develop evidence-based communication and behaviour change strategies that support people to address unhelpful beliefs and emotions and strengthen coping abilities as the UK moves through and beyond the SARS-CoV-2 pandemic.

**Supplementary Information:**

The online version contains supplementary material available at 10.1007/s12529-022-10055-z.

## Introduction 

The UK government imposed national restrictions (lockdown) on 23rd March 2020 in an attempt to limit SARS-CoV-2 transmission [1], causing significant disruption to the UK economy, public systems and people in society [2]. Little is currently known about the thoughts, feelings and coping behaviours of people in the UK towards this novel health threat.

The Common-Sense Model of Self-Regulation (CSM) states that beliefs about the identity, cause, controllability and curability, consequences and timeline of a health threat drive emotional and behavioural reactions to it [3]. Individual beliefs about SARS-CoV-2 are therefore likely to affect coping with the threat and compliance with public health advice.

Several UK studies have demonstrated the psychological impact of the first wave of SARS-CoV-2 infection [4–6]. Increases in common mental health disorders, mainly anxiety [7] and depression, were seen in people with existing mental and physical health conditions (EHCs) compared to before the pandemic [5]. This is concerning given that negative emotional reactions are associated with poorer coping [8].

Higher rates of behaviours related to obesity were seen in some people during the pandemic [9]. Poor health and behavioural outcomes are more common in people with EHCs [10] who are also at higher risk of morbidity and mortality from SARS-CoV-2, as are people who smoke or who are overweight or obese [11, 12].

Many studies to date indicate *how* people reacted to the threat of SARS-CoV-2, but do not explain *why* psychological responses vary, with few exploring people’s views and experiences. Ogueji et al. [13] found that a mixed sample of UK residents performed adaptive and maladaptive coping strategies, including health protective and health-threatening behaviours, such as socialising with loved ones and consuming alcohol (respectively), but failed to identify key underlying beliefs. No studies have compared people with and without EHCs. Other studies have indicated that personal risk perception [14] and trust in politicians may affect behavioural responses [15]; their focus however was adherence to social distancing and isolation interventions, not health behaviours. Understanding the beliefs underlying health threatening behaviours is paramount to supporting people, particularly vulnerable groups, to cope throughout the global pandemic.

Several studies have focused on vulnerable groups [16–21] and healthcare professionals [22–24] during the SARS-CoV-2 pandemic, but few have targeted people with EHCs. These studies show that the pandemic had a mainly negative impact on psychological and social well-being and daily functioning, but were specific to people with existing mental health difficulties [25] and respiratory conditions [26]. Another study reported a range of impacts of the global pandemic on the mental health and well-being of people with long-term conditions (LTCs) in the UK, particularly social isolation and healthcare access and delivery, but did not address implications on coping behaviour [27].

This study aimed to investigate how the threat of SARS-CoV-2 influenced the thoughts and feelings of people with and without EHCs in the UK and the subsequent impact on health-protective and health-threatening coping behaviour.

## Methods

### Design

The design is a cross-sectional online survey with the option to add additional free-text comments to specific questions. The survey yielded a large amount (*n* = 3186) of rich free-text responses detailing participants’ views of the threat of SARS-CoV-2 and their experiences of the first ‘wave’ of SARS-CoV-2. Such detail is often limited or absent in survey studies. This study therefore presents the valuable contributions of participants in a systematic, structured way that is consistent with the requirements of qualitative analysis [28].

### Participants

The participants were adults aged 18 years and over who live in the UK.

### Materials

An online survey was developed for this study. The survey was split into four sections. Section 1 gathered participant demographic information. Sections 2 to 4 comprised questions related to personal beliefs, emotions and behaviour towards the threat of SARS-CoV-2, respectively.

Survey items were based on some, but not all, concepts from existing dominant theories and models of responses to health threats. The CSM [3], the Transactional Model of Stress and Coping [29], the Health Belief Model [30, 31] and Protection Motivation Theory [32] guided survey development, though we compromised on the number of questions included in an effort to prevent social response bias and questionnaire fatigue. For example, no specific questions relating to causal factors [3] were included. A combination of complementary theories and models was favoured as each is particularly suited to examining either cognitions, emotions or coping responses [33]. Each survey section included an optional question with a free-text response box in an attempt to elicit additional information from participants about their beliefs, emotions and coping behaviours. These were generic questions and so were not mapped closely to any theory/model. Table 1 (see Supplementary material) maps the survey against their associated theoretical models and constructs.

The survey was hosted on Jisc, an online survey platform. To ensure data were captured in real time, the survey was not piloted prior to launch.

### Procedure

This study was approved by the Research Ethics Committee in the School of Healthcare Sciences, Cardiff University (reference: REC723). Snowball-sampling techniques were used to recruit participants. The survey was shared on the websites and social media platforms of Cardiff University (CU), Hywel Dda University Health Board (UHB) and HealthWise Wales (HWW); a database of health research participants living in Wales. The survey URL was also shared with existing contacts via email, WhatsApp and social media platforms including Twitter, Instagram, and Facebook. Informed consent was obtained prior to participants completing the survey. The survey was open from 8th April to 14th June 2020.

### Analysis

Anonymous free-text data were imported into NVivo. A framework analysis [34], guided by concepts of the CSM, was conducted to identify themes across the data. Data were independently and deductively coded by three authors; JC coded data that captured beliefs about SARS-CoV-2, LN coded data reflecting people’s emotional reactions to it, and RH coded data that described health behaviours that people performed to cope with the health threat. New codes were identified for data that was relevant to the study aims but was separate from the CSM. Any similarities and differences between people with and without EHCs were noted.

To ensure the data were coded consistently, one author (SA) independently coded a sample of free-text responses. Another author (SJ) checked the sample of coded free-text responses against the original codes and reviewed the authors’ comparisons between people with and without EHCs. Any discrepancies were discussed and resolved by the research team.

One author (RH) charted relevant codes against selected concepts from the analytical framework (see Table 2, Supplementary material) and scrutinised the data for overarching themes and sub-themes. The research team subsequently reviewed, refined and defined the themes.

## Results

Approximately 9110 people in the UK, including 4377 with one (48%) or more (10%) EHCs, responded to the survey, and 2763 respondents provided at least one free-text response. Sample characteristics of the participants who provided at least one free text response are reported in Table 3 in Supplementary information.

Three themes were derived from the free-text response data: (1) *locus of control* plus the sub-themes (1.1) *internal locus of control* and (1.2) *external locus of control*; (2) *emotional impact* including the sub-theme (2.1) *vulnerable groups* and (3) *coping strategies* comprising the sub-themes (3.1) *health-protective behaviours*, (3.2) *health-threatening behaviours*, (3.3) *social connectedness and context* and (3.4) *need for a collective approach to coping.*

### Locus of Control

This theme describes the extent to which people felt in control during the first wave of the SARS-CoV-2 pandemic and their perceptions about who and what were responsible for it.

#### Internal Locus of Control

Most people experienced a loss of personal control during the pandemic: “isolation… everything has been taken away from me.” (812, female, 61–70 years, hypertension, osteoporosis). Many recognised the importance of focusing on aspects of life that they could influence to regain a sense of control within the broader, mostly uncontrollable situation:

We should concentrate on those things that we can control, not to worry unduly about those that we cannot. (907, female, 31–40 years).

#### External Locus of Control

The pandemic was largely attributed to a range of factors beyond peoples’ personal control. Some, particularly those with EHCs, thought SARS-CoV-2 was man-made in China for economic benefit or a natural event:

It’s part of nature’s cycle, science will understand it and control it until another pandemic comes along. (614, female, 61–70 years, Asthma)

Some participants held God accountable for the pandemic and others believed “God is in control” (4619, female, 31–40 years, Asthma) of overcoming it.

Several participants believed “this [pandemic] was always going to happen” (211, female, 31–40), reasoning that socio-political issues, including austerity measures and struggling health systems, affected how prepared the UK was to manage serious health threats.

The UK government was viewed as responsible for controlling the spread of and leading the public response against SARS-CoV-2. However, some criticism was reserved for different approaches by the UK and devolved governments, which, for some with EHCs, negatively impacted coping. The political response was perceived too slow and driven by the economy rather than public health. Governments were accused of media meddling, which increased scepticism, anger, anxiety and decreased trust in politicians.

Thank heavens for devolution. The UK government in London have blood on their hands. The Welsh government have made mistakes too but I trust them more. (4344, female, 61–70, TSDM, chronic kidney disease)

I believe we are being lied to about the origin and extent of this virus. One thing that is causing me a huge amount of distress is the moving goalposts of the “peak” of the virus… It makes me feel extremely hopeless and out of control. (919, female, 31–40, mental illness)

Frustration was also felt towards people, especially younger generations, who were seen not to be taking individual action to limit the spread of SARS-CoV-2 by following government guidelines or restrictions. Public compliance was seen to depend on the perceived seriousness of SARS-CoV-2:

I wish the “invincible” age group would take it more seriously. The 18-25 year olds. I wish people would in general take it more seriously. Be mindful of distancing. I am missing my family. I’m scared as a person who is shielding yet my husband still has to go to work. I wish that people understood the threat more. (1053, female, 41–50, cancer remission)

### Emotional Impact

Many people had difficulty managing their emotions effectively and experienced a “rollercoaster of emotions day to day” (731, female, 31–40) during the first national lockdown. A loss of control caused many to feel anxious:

I feel very anxious, and as though the things that will make the biggest difference in the response to this threat are the things I have least control over. (900, female, 31–40)

Feeling bored was cited as a health risk factor:

Very confusing time…having difficulty stopping eating convenience food when bored in the house. (1263, female, 51–60, T2DM)

#### Vulnerable Groups

People’s social context also affected individual coping abilities. People with EHCs and their significant others, people who lived alone, HCPs and working parents seemed to be more vulnerable to negative emotions and poorer coping.

People with EHCs were often concerned by media messages about health and access to healthcare and were fearful of reintegrating into society:

I’m too afraid to go out. I feel fairly safe at home. I do not want to think about returning to normal as it scares me. (2982, female, 61–70, gallstones, obesity)

They felt less able to cope; individuals found it difficult maintaining concentration and motivation to work, exercise and perform everyday tasks.

People who lived with and cared for vulnerable individuals were also fearful of leaving the house and felt restricted and guilty for not being able to support others:

I don’t live alone, isolated due to shielding partner, just exercising, so my choices are limited. (1845, female, 71–80)

Hyper vigilant about leaving house myself and kids as partner is shielded...find it exhausting. (1776, female, 41–50)

Concern about the impact of social isolation on the mental health of people who lived alone was widespread:

Being alone is hard. I miss physical presence of loved ones (1678, female, 71–80).

Although this was not the case for everyone who lived alone:

I live alone and work freelance from home. It has made little difference, except to my social life. (2192, female, 51–60, hypertension)

Many HCPs continued working as usual, though some worked longer shifts during the pandemic, which deprived them of time and energy to engage in leisure activities. They were anxious about the availability of PPE.

Busy with work in NHS, currently working around 60 hours, additional hours as too busy to leave. Not registered or paid for. (175, female, 18–30)

Adults with and without EHCs, who were managing jobs, childcare and home schooling simultaneously, experienced frustration and guilt relating to lacking productivity. They had little time or energy for themselves:

I have two young children who I am now sole carer and teacher for. I have full responsibility for their care (eating/dressing/washing), well-being, development and schooling. I have no time in my day for activities outside of them […] I have no time to collect my wits or for my own well-being. (2258, female, 31–40).

### Coping Strategies

Individuals adopted different strategies in order to cope with the threat of SARS-CoV-2. Many people spent less time commuting to work due to lockdown, which afforded more time with family and for meaningful activities.

#### Health-protective Behaviours

Gathering information about SARS-CoV-2 and adhering to social distancing and self-isolation guidelines were considered important for coping. Performing health-protective behaviours, such as healthy eating and physical activity, were perceived to benefit mental health and coping overall:

Exercise has been a big part of my way of dealing with these circumstances. (806, female 41–50)

Some people with and without EHCs reported regular participation in physical activity prior to the pandemic. People who usually played sports reported being less active due to facility closures, government restrictions and shielding. Other barriers to exercise included fatigue and low motivation:

I normally go to the gym 3-4 times a week but physical exercise has been a challenge since lockdown and I think I may well be the size of a house when we finally reach daylight. (495, male, 41–50)

Some with EHCs were less sedentary and more motivated to be physically active:

I have made an effort to take a walk most days to get out of the house. Normally I’m very sedentary so actually getting more exercise than usual. (154, female, 51–60, T2DM)

There seemed to be a bi-directional relationship between these health-protective behaviours and mood. For some, “initial good habits have tailed off as I become lethargic” (4438, female, 31–40, asthma). Others reported reduced engagement in health-threatening behaviours over time:

I started lockdown eating quite unhealthy foods, lots of treats. But in the last week or so I am eating more healthily as it was affecting me physically and mentally in a negative way. (1431, female, 31–40)

Performing psychological techniques facilitated coping: “I use meditation and yoga to relax.” (5058, female, 51–60). Popular practices included mindfulness, gratitude and positive reframing. Many people, mostly those with EHCs, found solace in religion: “My faith is important, but I do not believe it protects me from possibly getting Covid-19” (2551, female, 71–80, cancer)

Planning and maintaining a daily routine, and keeping busy with new or existing hobbies, including housework and gardening, were recognised as supporting well-being:

My psychological health is maintained by having initiated a structured daily routine from the very beginning of the lockdown. The routine addresses physical, emotional, social and cognitive well-being (2961, male, 61–70 year, HWW)

Such activities fostered a personal sense of control, purpose and achievement:

I try to keep busy every day […] to feel that I have achieved something every day — no matter how small. (2287, female, 51–60)

#### Health-threatening Behaviours

Some people employed avoidance coping and individuals experiencing mental illness reported performing more health-threatening behaviours at the beginning of the national lockdown:

I did get stuck in a rut with eating, drinking and not exercising but now I have started eating well, drinking less and exercising for an hour a day. (2964, male, 31–40, mental illness)

#### Social Connection and Context

The lack of social connection during lockdown was a major challenge, with many reporting that they were “missing family more than usual” (220, male, 61–70, heart and lung disease).

Regular communication with others was vitally important for coping. Staying connected with and supporting others through voluntary activities gave people a sense of purpose and an active role in fighting the threat:

I’ve found having a structure to my day has helped so I have a plan that I stick to […] Started volunteering locally once a week from start of lockdown as wanted to feel like I was doing something to make a difference. (2111, female, 41–50)

However, people with EHCs were often denied, or were unable to, continue volunteering due to their high-risk status, which caused frustration. This was also the case for others in their household.

The social context also affected coping. Living in a rural area and having access to a garden or green spaces was perceived to improve psychological well-being, as were good weather and owning a pet:

My little dog is a wonderful companion and makes sure I exercise each day. (537, male, 71–80, cancer)

Conflicting opinions on the use of social media during the pandemic were expressed by many:

Torn between finding out the news and info on corona and switching off from it. It is a balancing act. (1052, female, 61–70, osteoporosis).

Some said it was vital for staying connected to others and reducing feelings of loneliness:

Social media is important to me, I am able to catch up with friends and family. I don’t feel quite so isolated. (12, female, 61–70, hepatitis).

For some with EHCs, virtual communication was instrumental for strengthening existing and forming new relationships:

Through this [WhatsApp] I have actually got to know more people in the village, a good thing. (3649, female, 51–60, asthma).

Reducing screen time and restricting social media and news consumption were necessary health-protective behaviours: “I’ve reduced social media time and reading news because it stresses me out” (72, female, 18–30, mental illness).

#### Need for a Collective Approach to Coping

The pandemic was labelled a learning opportunity and a time to reflect on current lifestyles and “pause for thought about life on Earth in general” (2907, male, 71–80, asthma, stroke):

I hope we will all appreciate the time to reflect on our previous “business”, sometimes unnecessary, and to appreciate the smaller simple pleasures that cost nothing such as family walks looking at nature […] Time to be kind and considerate […] and to re-evaluate what is most important in life. (469, female, 71–80)

Collective action between the government, health service, researchers and the public was deemed essential to control the acute threat, prevent anticipated spikes in infection rates and overcome the perceived lasting adversities of SARS-CoV-2, specifically poor mental health and widening social inequalities. People believed that national collaboration would better prepare the UK to cope with future health threats effectively and a shift from individualism to collectivism would change society for the better:

A very hard way to learn that we cannot keep going along as we have been. Something was going to happen and it could have been far worse. Cooperation, help and kindness are some of the many positives the world has shown so let us hope this continues in the aftermath. (703, female, 61–70).

## Discussion

People with and without EHCs experienced a personal loss of control during the first national lockdown. Uncertainty and doubt in the ability of politicians of the four nations to lead the UK through the pandemic was widespread. Differing political responses and negative media portrayals of events elicited mostly feelings of anxiety, depression and anger, which challenged coping resources. Working parents, HCPs, people who lived alone and at-risk individuals and their relatives found the rapid adjustments to life and work particularly difficult and distressing because they had no control and little to no support. Perceived severity of SARS-CoV-2, mood and motivation all influenced engagement in health behaviours. Activities that instilled a sense of control and purpose were important for self-management. A collective societal approach was deemed necessary for overcoming the short- and long-term effects of the global pandemic.

### What the Present Study Adds

As in previous studies, we show that concerns about disjointed political responses [26] and personal risk perception [15] influence emotional reactions and compliance with public health behavioural strategies. Building on the earlier finding that levels of perceived control differ between individuals [35], we found a strong internal locus of control improved self-management of negative emotions brought about by political and media reactions. We emphasise the importance of health-protective behaviours that instil a sense of purpose and achievement, including exercising, healthy eating and psychological practices, and highlight that negative emotions, feelings of anxiety, boredom, fatigue, loneliness and low motivation reduced willingness to perform adaptive coping strategies.

Similarly to Fisher et al. [27], this study indicates that people with EHCs may be fearful of reintegrating into society following prolonged social isolation. This is concerning as fear responses could fuel avoidance coping strategies and reinforces calls for increasing psychological support for high-risk individuals [36]. Furthermore, many partners, relatives and friends experienced a loss of personal freedom and cognitive dissonance due to living with at-risk individuals. These groups may require additional support.

### Strengths and Limitations

This study is one of the first in the UK to analyse qualitative data exploring the real-time beliefs, emotions and behaviours of people with and without EHCs towards the threat of SARS-CoV-2. Unlike earlier studies [17, 26], we recruited a broad sample of people to our survey and so the findings reflect the voices of different people across the UK. Our data offers rich insight into the lived experiences of SARS-CoV-2 and psychological factors, specifically health behaviours, to address in order to support people in the UK to cope effectively throughout the pandemic. This level of qualitative detail is often missing from survey studies, so the volume and quality of the present findings are major strengths of this study. As is the contributions of a Patient and Public Involvement co-investigator (LN).

The use of an established theoretical model to analyse the data ensured the transparent and systematic analysis of the free-text survey responses [37] and strengthens this study further. Using the CSM allowed for a thorough analysis of coping strategies and the capture of a number of underlying cognitive, emotional and social factors that could form the basis of behaviour change interventions to improve public responses to SARS-CoV-2 and future health threats.

As the survey adopted a cross-sectional design, we were unable to capture changes in people’s responses to SARS-CoV-2 overtime, though this would have been challenging to investigate given the frequent changes in government policy. Despite the diversity in our sample, a large proportion of respondents were recruited from a database of health research participants living in Wales. The views and experiences represented may disproportionately reflect those of people based in Wales who are concerned about health, limiting the transferability of the findings.

### Implications for Research and Practice

SARS-CoV-2 is likely to remain a threat to people’s health until a vaccine becomes widely distributed and the virus spread better controlled. Replicating this study to understand how people’s thoughts, feelings and coping behaviours change over the course of the pandemic in response to subsequent national and local lockdowns could inform how governing bodies support people to cope effectively in the event of future health crises.

Our sample regarded lockdown as a time for learning and development and so, it may be a ‘teachable moment’ for health behaviour change [38] with appropriate support. Further qualitative research could identify personal barriers and facilitators to performing health-protective behaviours and determine the level of dedicated psychological support required to enable vulnerable groups, including at-risk individuals and HCPs, to cope effectively with the threat and impact of SARS-CoV-2.

However, information may not engender health behaviour change [39]. Behavioural scientists, experts in theory-based behaviour change approaches, could support people to self-manage their mood and motivation and plan for successful re-adaptation. Behavioural scientists possess extensive knowledge of theory-based communication approaches for behaviour change [40] and therefore could advise on framing health messages to facilitate compliance with public health policy, encourage positive coping and avoid sensationalism.

## Conclusion

The SARS-CoV-2 pandemic has profoundly affected the psychological functioning of UK residents, especially people with existing health conditions, HCPs and working parents. Interventions are needed to enhance people’s internal locus of control and empower individuals to engage in health-protective behaviours that help to buffer the psychological burden of SARS-CoV-2 and other variants. The current pandemic is a unique opportunity for UK leaders to collaborate with behavioural scientists to deliver clear public health messages and develop strategies that address inaccurate beliefs, minimise anxiety and promote population-level health behaviour change to engender active coping responses to SARS-CoV-2 and prepare people to readapt as the national lockdown and associated restrictions ease.

## Supplementary Information

Below is the link to the electronic supplementary material.

Supplementary file1 (DOCX 33 KB)

## Declarations

### Animal Subjects

This article does not contain any studies with animals performed by any of the authors.
