# Supplementary material for: Covid-19 Coping Survey: an In-depth Qualitative Analysis of Free-Text Responses from People With and Without Existing Health Conditions in the UK
Source: Int J Behav Med. 2022 Feb 7;29(6):743–51. doi: 10.1007/s12529-022-10055-z (PMC8821867; doi:10.1007/s12529-022-10055-z)
Supplement: Supplementary file 1 — Supplementary file1 (DOCX 33 KB) [file 12529_2022_10055_MOESM1_ESM.docx]

**Supplementary Material**

| Table I. Covid-19 coping survey items mapped to underlying theoretical models and constructs | | | |
| --- | --- | --- | --- |
| **Moral / Theory** | **Theoretical Concept** | **Survey Items** |  |
| **Common-Sense Model of Self-Regulation [3]** | **Identity (perception of threat)** | I believe this is a real threat to mine or my families health  I believe this is a real threat to my or my family’s well-being  I am confident that this threat will not affect me or my family |  |
|  | **Controllability - locus of control** |  |  |
|  | ***External Locus of Control*** | I believe scientists will find a solution to this  I believe politicians will get us through this threat  I believe Doctors / healthcare staff will get us through this threat  I believe what will be will be and I cannot influence things at all  I believe my faith will get me through this threat |  |
|  | ***Internal locus of control*** | I think it’s important to focus on what I can do for others during this threat |  |
|  | **Consequences** | I believe something positive will come from this threat  I think things are never going to be the same again  Emotional Consequences:  I am worried for me or my family now  I feel confused about how I feel  I am worried for my or my family’s future  I feel down or depressed about this threat  I am concerned about spending so much time on my own  I am concerned about spending so much time with my family  I feel angry about this threat  I feel guilty about this threat  I feel optimistic for the future beyond this threat  I feel energised in response to this threat  I don’t feel anything different to usual I feel numb or unable to feel anything I feel worried about my health |  |
|  | **Timeline** | I believe this is a short term threat  I believe this is a long-term threat |  |
|  | **Cause** | N/A |  |
| **Health Belief Model**  **[25, 26]** | **Perceived susceptibility** | I believe this is a real threat to mine or my family’s health  I believe this is a real threat to my or my family’s well-being  I am confident that this threat will not affect me or my family |  |
|  | **Perceived severity** | I believe this whole thing is exaggerated |  |
|  | **Perceived benefits** | I believe something positive will come from this threat |  |
|  | **Perceived barriers** | N/A |  |
| **Transactional Model of Stress and Coping [24]** | **Primary appraisal (significance)** | I believe this is a real threat to mine or my family’s health  I believe this is a real threat to my or my family’s well-being (mental health)  I am confident that this threat will not affect me or my family  I believe this whole thing is exaggerated  I believe this is a short-term threat  I believe this is a long-term threat  I believe something positive will come from this threat |  |
|  | **Secondary appraisal (personal resources/ options)** | I believe my faith will get me through this threat  I think it’s important to focus on what I can do for others during this threat  I believe what will be will be and I cannot influence things at all |  |
|  | **Coping responses** |  |  |
|  | ***Active coping*** | I am focusing on finding the positives every day, more than usual  I have taken the initiative to reach out to others physically (e.g. volunteering or caring for neighbours), more than usual  I have taken the initiative to reach out to others virtually, more than usual  I have been physically active, more than usual  I have kept to a structured timetable for everyday activities, more than usual  I have been practicing psychological techniques such as mindfulness or yoga, more than usual  I have decided to learn everything I can about this threat  I have taken steps to stay healthy and fit, more than usual  I have been doing active work in my community (that could be work community; locality or family or friendship groups), more than usual  I have been spending time with family physically, more than usual  I am keeping busy with practical, everyday living or work tasks, more than usual I am working at my job, more than usual |  |
|  | ***Avoidance coping*** | I am finding it difficult to create any structure to my day, more than usual  I am finding it difficult concentrate on physically doing anything, more than usual  I am taking over the counter medication / tablets, more than usual  I am taking prescription medication / tablets, more than usual  I have been passive, more than usual  I have been drinking alcohol, more than usual  I have been smoking, more than usual  I have been taking drugs, more than usual  I have been eating unhealthy food, more than usual  I have been less physically active than usual  I have decided the best thing is to stop thinking about it completely |  |
| **Protection Motivation Theory [27]** | **Perceived severity** | I believe this whole thing is exaggerated |  |
|  | **Perceived vulnerability** | I believe this is a real threat to mine or my family’s health  I believe this is a real threat to my or my family’s well-being (mental health)  I am confident that this threat will not affect me or my family |  |
|  | **Perceived self-efficacy** | N/A |  |
|  | **Perceived efficacy of recommended preventative behaviour** | N/A |  |
|  |  | **Optional Free-Text Survey Items^a^** |  |
|  |  | If there is anything else you wish to tell us about your thoughts/beliefs in relation to the threat, please write below. |  |
|  |  | If there is anything else you wish to tell us about how you feel in relation to the threat, please write below. |  |
|  |  | If there is anything else you wish to tell us about your behaviour/actions in relation to the threat, please write below. |  |

^a^These questions were generic and so were not mapped closely to any theory/model.

| Table II. A summary of codes mapped onto the concepts of the Common-Sense Model of Self-Regulation [3] | | | | | | | | |
| --- | --- | --- | --- | --- | --- | --- | --- | --- |
| **Cause** | **Coherence** | | **Controllability/ Cure** | | **Identity** | **Social context** | | **Timeline** |
| Inevitability of pandemic due to:   - Austerity measures - A socio-political problem - Past occurrence and responses to viruses e.g. SARS - Treatment of animals and natural environment/lack of veganism - Lifestyle and behaviour - Karma for ‘human crimes against nature’ - Nature’s response - Natural selection   *Anger/frustration:* (**move to coherence?)**   - *China* - *Researchers from China/ USA* - *‘Man-made’ virus and conspiracy theories*   Other causes:   - Poor international health system standing - Early transmission of virus in UK – pre. 2020 – link to slow UK lockdown   Negative impact of media dissemination:   - Fearmongering - Sensationalism | *Bravery of key workers*  Anger:   - Lack of individual responsibility in society - “Common sense” approach not working - Lack of social distancing   Anger/frustration:   - Poor communication from media and government. - Mixed messages re: guidance/regulations - Lack of transparency - Misinformation - Media interference - Exploitation of pandemic – business and media - Blanket rules across UK/lack of nuanced/targeted approach - Link to poorer mental health and wellbeing - Lack of government planning PPE; Lack of lessons learned from previous pandemics   Slow lockdown   - All nations approach: Problems with message clarity and coherence   Exaggeration of threat leads to creation of fear in society | | Internal  Restricting news input  Social distancing and self-isolation:   - No strangers - No family - No unnecessary travelling   Stockpiling medication  Strict regimes to minimise infection, e.g. Infection control in house  Positivity  Structure  Working more  Positive health behaviours:   - Healthy eating - Exercise - Vitamin supplements - Following scientific guidelines   Research/information gathering  Common sense approach  **What do we need to do?** *Need for vigilance/precautions- individual level*   - *Collective responsibility* - *International collaboration* - *Trust in Government approaches/’the science’*   Internal/external  Collective action Importance of social awareness –absolutely – lots about strength of solidarity and collaboration  External  Immunisation – faith that scientists will find a cure  Border control:   - People - Products   Increase NHS funding and capacity  *Inevitability – lack of controllability*   - *Epidemiological context* - *Feeling helplessness*   *(Move to coherence?)*  *Faith in healthcare workers to get society through pandemic  Bravery of key workers*  Society powerless to prevent | | Loss of ‘physically active’ stat Positive health behaviours:   - Healthy eating - Exercise - Vitamin supplements - Following scientific guidelines   us:   - Shielding – new status of ‘vulnerability’ - Lack of sports/   opportunities for exercise  Religion – protective factor?  Humanism  Mental health and illness:   - Mental strength – stoicism and resilience - Having experienced previous chronic health problems   Introversion   - Better outcomes for introverts? More prepared for lockdown…   Employment status   - Unemployed - Retired - Furloughed - Key worker - link to pragmatism (coherence)   *Shielding/vulnerability*  *Healthcare workers - brave*  **Identity of virus:**  *Underestimation of covid-19 threat*  Perceived severity of threat of virus (less serious associated with less compliance with government guidance)  Many perceived the pandemic as a ‘learning opportunity’ for society  Opportunity   - Re-evaluate how we are living - Solidarity /working collectively - Overcoming together - Empowerment - Courage | **Societal factors affecting coping:**  Socio-geographic context as protective factor:   - Rural/larger garden – viewed safer – living in ‘*cocoon*’ - Link to positive mental health - Lack of access to green spaces – risk factor - Air pollution- risk factor   Cohesive community as protective factor – and pets?  **Political factors (sub-theme):**  Negative responses   - Party politics   *Government* approaches (link to coherence? – anger/frustration)   - *Misinformation (many people felt anger/anxiety about the truth in the breadth of information available* - *Slow response to lockdown* - *Incompetent* - *Policy hyperactivity* - *Economy-driven* - *Failure to protect public* - *Failure to protect key workers* - *Chronic underfunding of NHS* - Government is perceived to be self-centred – concerned by finances more than health - Lack of political collaboration/consensus - Political ‘meddling’ in the media - Perceived return to status quo/ lack of learning   Conservative Party:   - Unethical - Ideologically driven   Devolution   - Confusion - Conflicting messages - Need for all nations approach - Welsh government focus on south Wales to detriment of other regions | | Long-term impact   - Clear information required for management of time - Stockpiling food and medication - Impact on mental health (negative) - Living in limbo - Uncertainty of recovery/vaccine - Vaccine needed or inevitable contraction of virus will occur - Inevitable return to status quo – how key workers are valued, lifestyle, behaviour pre virus   Hope   - More appreciation for key workers - Health of population – physical - Positive societal change   *Coping* (link to consequence/behaviour)   - *Return to simpler times* - *One day at a time approach* - *Slower pace of life* - One health worker had the perception that spikes in coronavirus cases is more than likely, even if a vaccine is made available (pessimism about the future). Highlights cyclical timeline |
| **Consequence** | | | | | | | | |
| **Behaviour** | | **Society** | | **Emotions** | | | **Other** | |
| Protecting family and friends, the public   - Checking in and contacting - More community time - More time family and friends - Advocacy – educating others to comply with lockdown rules   Increased phone usage  Increased consumption of TV/media/government briefings, but, decreased consumption of social media/TV/government briefing  Behaviour as normal   - Those living alone - Key workers/front line staff. Some key workers felt unable to maintain their exercise regime due to increased working hours   Following advice government  social distancing  Being in a ‘vulnerable’ category – feeling more confined/trapped/limited  Living with someone in vulnerable category  Experience of emotional/financial abuse  Working more  More responsibility   - Partner care - Childcare - *Home schooling* - *Eeducating others about how to minimize risk of transmission/contraction/comply with lockdown rules and providing truthful information (advocacy). *Especially evident in healthcare staff (nurses)*   *Active* Coping strategies   - Seeking information/doing research - Religion/faith - Taking up new hobbies/learning new skills, or activities that people did not have time for prior to lockdown - Keeping calm (e.g. taking one day at a time - thinking in the present rather than long-term) - Brain training - Sports - Increased physical activity - Increased sexual activity - Structure/routine seeking - Psychological techniques/interventions e.g. *Mindfulness* - Healthier eating - Appreciating the natural environment - Taking responsibility – childcare - Creativity - Supporting others/community work - Social support - Celebrating small achievements   Pets seem to facilitate positive coping (e.g. physical activity) and buffer psychological discomfort/distress in the UK.  *Avoidance coping strategies*  Distraction techniques:   - Housework/ gardening - Animals - Denial   Unhealthy lifestyle behaviours: - Stress eating   - Hard to keep fit/active - Increased alcohol consumption   Dissociation | | **Pandemic widening Inequalities:**   - Financial - Disability and chronic illness - Race and ethnicity - Age - Mental health   **Inter-family/ community isolation**:   - Link to negative effect on mental health   **Economy/finance:**  Financial vulnerability and increased effects   - Marginalisation/widening inequalities   Financial uncertainty and anxiety   - Deteriorating savings - Businesses and jobs - Furlough and the fear of redundancy - Rising cost of goods - Link between finances and mental health - Austerity   Greater investment in science required | | Negative emotions:  Anxiety/fear:   - Increasing use of technology/consumption of media/TV - Work - Overwhelmed by media/ ‘fake’ news - Transmitting the virus - Death/serious illness - Living alone - Lack of PPE – key workers - Frightened to leave house - Frightened about second/third waves - Health of family/community members, especially those living abroad - Nothing will change/ lessons won’t be learned - Uncertainty is scarier than the virus itself - Politics takes priority over health - Uncertainty of life   Depression   - Health status - Future/ long virus timeline - Link to time in lockdown   Frustration/anger (link to coherence)   - Slow lockdown - Government advice - Self-centredness of government - Conflicting messages – government and media - Fluctuating emotions – ‘*rollercoaster days’* - Self-isolation - Restrictions on day to day life - Inability to help/volunteer due to vulnerability/health status - Lack of PPE - Others’ behaviours – lack of mask wearing/lack of social distancing - Problems with maintaining healthy work/life balance - Childcare/education/work demands - Confusion as to best way to approach lockdown - Irritation - Indecision paralysis - Pandemic highlighting and exacerbating existing inequalities - Change of life plans – marriage etc. - Helplessness - Feeling trapped/confined (particularly vulnerable people) - Feeling unprotected/abandoned by the system - *Less patience* - *Sacrifice of lifestyle* - *Struggling to establish working from home routine* - *Responsibility/ethics/morals/behaviours of others* - *Needs ignored by Government*   Mistrust/ Scepticism   - Mostly in politicians - The media   Shock – at the beginning   - Speed of change (negative)   Guilt   - Feeling fit/exercising - Lack of involvement – volunteering/helping - Not exercising - Lack of productivity at work due to childcare/home education demands - Lack of parenting ability due to work/other care demands - Lack of bravery - Inability to help - About the environment   Loneliness   - Living alone   Sadness, grief and loss   - Covid death toll - Losing loved ones   Apathy   - Lack of motivation - Loss of interest - Lethargy - Torpor - Boredom   Regret (often linked to the inability to help/volunteer more)   - People will not learn from this situation   **Positive emotions:**  Enjoyment/gratitude   - Peace in lockdown - Better structure - Lack of commute - More downtime - More relaxed about appearance - Working from home positives - More time with family - Access to internet - Introversion - Key workers - Less pollution - Increase in altruism   Acceptance   - Reality of situation (+ve) - Resignation (-ve) - Lack of worries   Resilience   - Link to lack of empathy – ‘*manning up’* - Pragmatism - Patience - Strength   Optimism and hope   - The pandemic will change society for the better   Faith that scientists will find a cure | | | - Need to be more self-conscious/ aware of the impact of lifestyle on the environment and other humans - Greater understand of human beings (positive and negative traits, best/worst in people) - Increase collectivism (sense of society), decrease individualism - Recognition of the importance of need for human interaction/physical contact (e.g. hugs) | |

| Table III. Sample characteristics of participants who provided at least one free text response | | |
| --- | --- | --- |
|  | *n* | (%)^a^ |
| **Total** | 2,763 |  |
| **Survey** |  |  |
| [organisation I] | 872 | (31.6) |
| [organisation II] | 1,888 | (68.3) |
| [organisation III] | 3 | (0.1) |
| **Country** |  |  |
| *England* | 15 | (0.8) |
| *Wales* | 1,901 | (99.1) |
| *Scotland* | 3 | (0.2) |
| *Other* | 844 | (30.6) |
| **Age (Years)** |  |  |
| *18 – 30* | 185 | (6.7) |
| *31 – 40* | 303 | (11) |
| *41 – 50* | 365 | (13.2) |
| *51 – 60* | 551 | (19.9) |
| *61 – 70* | 803 | (29.1) |
| *71 – 80* | 499 | (18.1) |
| *81+* | 53 | (1.9) |
| *Prefer not to say* | 4 | (0.1) |
| **Gender** |  |  |
| *Male* | 862 | (31.2) |
| *Female* | 1,895 | (68.6) |
| *Prefer to self-describe* | 4 | (0.1) |
| *Rather not say* | 2 | (0.1) |
| **EHCs** | 4377 | (48) |
| *Cardiovascular* | 276 | (10) |
| *Respiratory* | 363 | (13.1) |
| *Diabetes* | 183 | (6.6) |
| *Cancer* | 80 | (2.9) |
| *Dementia* | 2 | (0.1) |
| *Mental illness* | 253 | (9.2) |
| *Pregnancy* | 17 | (0.6) |
| *Other* | 711 | (25.7) |
| *No EHC* | 1,304 | (47.2) |
| **Ethnicity** |  |  |
| *White/White British* | 2,655 | (96.1) |
| *Black/African/Caribbean/Black British* | 13 | (0.5) |
| *Asian/Asian British* | 32 | (1.2) |
| *Mixed/Multiple Ethnic Groups* | 28 | (1) |
| *Other Ethnic group* | 35 | (1.3) |
| **Highest qualification** |  |  |
| *Usual high school qualifications in your country at age 16 (e.g. GCSE, O-level)* | 336 | (12.2) |
| *Usual high school qualifications in your country at age 18 (E.g. AS level, A-Level)* | 213 | (7.7) |
| *A college or university diploma or degree* | 1,198 | (43.4) |
| *A higher degree or professional qualification (e.g. a Doctorate or Masters level degree)* | 847 | (30.7) |
| *None of these qualifications* | 90 | (3.3) |
| *Other* | 61 | (2.2) |
| *Rather not say* | 18 | (0.7) |
| **Normally occupied** |  |  |
| *Full-time* | 904 | (32.7) |
| *Part-time* | 474 | (17.2) |
| *Unemployed, seeking work* | 28 | (1) |
| *Unemployed, not seeking work* | 106 | (3.8) |
| *Full-time education* *or training* | 87 | (3.2) |
| *Part-time education* *or training* | 36 | (1.3) |
| *Volunteer* | 173 | (6.3) |
| *Homemaker* | 85 | (3.1) |
| *Retired* | 1,147 | (41.5) |
| *Rather not say* | 13 | (0.5) |
| ^a^Percentages were rounded to one decimal place |  |  |
